# Use of Digital Healthcare Communication to Improve Urologists’ Surveillance of Lithiasis Patients Treated with Internal Urinary Drainage Pre- and Post-COVID-19 Period

**DOI:** 10.3390/healthcare11121776

**Published:** 2023-06-16

**Authors:** Mihai Vintilă, Dan Mischianu, Octavia-Sorina Honțaru, Mihai Dobra, Alin Gabriel Sterian

**Affiliations:** 1 Faculty of Medicine, University of Medicine and Pharmacy “Carol Davila” Bucharest, 020021 Bucharest, Romania; mihai.vintila@drd.umfcd.ro; 2 Department of Urology, University of Medicine and Pharmacy “Carol Davila” Bucharest, 020021 Bucharest, Romania; dan.mischianu@umfcd.ro; 3 Department of Urology, University Emergency Central Military Hospital, 010825 Bucharest, Romania; 4 Faculty of Sciences, Physical Education and Informatics, University of Pitesti, Târgul din Vale 1, Arges, 110040 Pitesti, Romania; 5 Department of Public Health Arges, Exercitiu 39 bis, Arges, 110438 Pitești, Romania; 6 Center of Uronephrology and Renal Transplant Fundeni, University of Medicine and Pharmacy “Carol Davila” Bucharest, 020021 Bucharest, Romania; 7 Department of Pediatric Surgery and Orthopedics, University of Medicine and Pharmacy “Carol Davila” Bucharest, 020021 Bucharest, Romania; gabriel.sterian@umfcd.ro; 8 Emergency Hospital for Children Grigore Alexandrescu, 30-32 Iancu de Hunedoara Boulevard, 011743 Bucharest, Romania

**Keywords:** internal stents, obstructive urolithiasis, COVID-19, disruptive technologies, online communication

## Abstract

The COVID-19 pandemic has greatly affected lithiasis patients, which has led to an increase in the number of internal stents that have been installed. In this paper, two studies were carried out, a clinical study and a quantitative study. The aim of the first study was to evaluate the incidence and the prevalence of bacterial urinary colonization in patients with obstructive urolithiasis who needed internal stents implanted. In the second study, a multiple linear regression was created to identify the opinion of urologists regarding the importance of using digital technologies to improve the communication process. The result of the clinical study illustrates that the prevalence of urinary colonization in patients with internal stents carried out for obstructive urolithiasis was 35%, with this value being influenced by co-infection with COVID-19. The results of the quantitative study illustrated the fact that urologists are open to using new online technologies to facilitate communication with patients. The results have high importance for both doctors and patients, illustrating the main factors that have the ability to influence the communication process. The hospital managers should take into account the results obtained in this study when they choose to use certain online communication technologies with patients.

## 1. Introduction

### 1.1. The Use of New Communication Technologies in the Medical Field

According to the World Health Organization (WHO), e-Health is the use of information and communication technology in health and other related fields [1]. Information and communication technologies (ICTs) have also been introduced in the health sector, with many healthcare departments being positively influenced by their use. The use of ICT in health is a means of supporting this field of activity [2]. The use of various technological tools in medical facilities can significantly help employees, and especially nurses, as well as medical service providers in general, to improve the level of medical care provided to patients. These technologies contribute to improving the quality of services provided, as well as other evaluation indicators in this field of activity [3]. There is a wide range of information and communication technologies used to support and deliver healthcare. Mair [4] proposed four general areas to be included in the e-health system that include a variety of ICTs. These are management systems, communication systems, electronic decision support systems and information systems. Management systems enable the acquisition, storage, transmission and display of administrative or clinical activities related to patients, such as electronic health records (EHRs) [4].

The European Union states that applying eHealth to the whole range of professions in the health sector has the potential to break fresh ground to access healthcare and increase the overall efficiency of the health sector. According to the European Commission, eHealth and telemedicine are based on the freedom of movement of services applicable to healthcare through the Directive on Electronic Commerce. eHealth is an emerging field at the crossroads of three fields: medical informatics, public health and business. The World Health Organization (WHO) has defined eHealth as “the cost-effective and safe use of ICT in support of health, related fields and research” [5]. Telemedicine [6] is the provision of assistance services and medical information using innovative technologies, especially ICT, in situations where a medical professional and the patient (or two medical professionals) are in different locations. Telemedicine [7] covers only the curative aspect of the healthcare sector and includes any remote interaction between patients and healthcare professionals and between healthcare professionals, whether synchronous or asynchronous. A generally accepted definition in several European countries is the one offered by the European Commission: according to which, telemedicine has the role of improving prevention, diagnosis, treatment, monitoring, health management and lifestyle.

mHealth (mobile health) [8] is one of the components of eHealth that is based on technologies such as smartphones and digital assistants in order to provide information and care services in the medical field. Among the services provided, the following can be mentioned: health monitoring, disease reporting and useful information for a healthy lifestyle. The use of mHealth tools [9] can favor an increase in primary and preventive health services used, the collection of specific data, the establishment of a treatment plan and the timely submission of medical analysis results [10]. Digital tools specific to the medical field, such as electronic medical records, data registries, portable monitoring or reporting devices, electronic therapy and educational platforms have the potential to improve access to health and provide personalized services for patients [11]. Digital devices that integrate data into services and electronic support for decisions, resources and interventions improve patient–provider communication [11].

### 1.2. The Importance of Using Internal Stents in Urology

The use of internal stents has become a current practice in urology departments worldwide. Made of polyurethane or silicone, the internal stent is flexible and autostatic, with curved ends that allow it to be held between the kidney at its upper end and the urinary bladder at its lower end [12,13,14]. Regardless of the purpose of its insertion—preventive, curative, palliative or other indications, the internal stents must ensure the flow of urine from the kidneys to the bladder [15,16]. Once inserted, the time of indwelling of the internal stents may vary from a few days to a few months, depending on the indication. Its removal moment must be chosen carefully, considering that a too-short period can be ineffective, and a too-long period can lead to complications (calcifications, obstructions, etc.) [17,18,19]. Only a few studies have been performed for the evaluation of the incidence and prevalence of patients with bacteriuria and urinary colonization among internal stent carriers, regardless of their indication. Additionally, studies on urinary tract infections usually include all patients with significant bacteriuria, symptomatic or not, without actually separating infections from urinary tract colonization [20,21]. In complicated urolithiasis with complete obstruction and/or secondary infection, an endo-prosthesis may be temporarily required, whatever the surgical method subsequently approached [22]. An ultrasound-guided percutaneous nephrostomy would be performed in the case of physiological urinary drainage failure [23]. The bacteria that colonize ureteral endoprosthesis develop in colonies, adhere to the internal stents, multiply and secrete “slime” as an extracellular polysaccharide matrix, in which salts and urinary proteins are subsequently incorporated [24,25]. This biofilm favors the adhesion of germs, protecting them from defense mechanisms and microbial agents [26,27,28,29].

The COVID-19 pandemic has greatly affected the work carried out by urologists. During it, medical personnel were put in a position to solve complex problems that they had not faced until that moment. In addition, patients with kidney problems who became infected with the SARS-CoV-2 2 virus showed some additional reactions that required immediate intervention by physicians. The complexity of the urological problems that appeared during the COVID-19 pandemic in lithiasis patients, as well as the difficulty of communicating with them due to the restrictions imposed at the national level, led us to carry out a thorough analysis of the problems that appeared in patients treated with internal stents, as well as how communication with them could be improved, in order to reduce possible complications that may arise later, due to non-compliance with the deadlines for extracting/changing medical devices. The research included two studies, namely, a clinical study and a quantitative study. The clinical study aimed to identify the incidence and prevalence of urinary bacterial colonization in patients with obstructive urolithiasis treated with internal stents during the COVID-19 pandemic. The second study had, as its starting point, the clinical results obtained following the conducting of the first clinical study, as well as physicians’ opinions regarding the communication problems that arose during the COVID-19 pandemic between patients and physicians due to the restrictions imposed at the national level. Based on these results, a quantitative study was carried out that had the role of determining the perception of Romanian urologists regarding the role and importance of the use of new technologies in terms of improving the communication process with lithiasis patients pre- and post-COVID-19 period.

## 2. Materials and Methods

### 2.1. Clinical Study

#### Clinical Study Design and Research Sample

The clinical study was carried out in the Urology Department of “Dr. Carol Davila” Central Military Emergency University Hospital, between January 2020 and June 2022 (the hospital was not assigned as a COVID-19 support hospital), using a batch of 212 patients. The inclusion criteria were as follows: any patient with an indwelled internal stent for obstructive urolithiasis, who gave his/her consent to participate in the study whatever their sex, age, comorbidities and complications. Patients who did not agree to participate in the study and pregnant women were excluded (there is no integrated Obstetrics—Neonatology department within “Dr. Carol Davila” Central Military Emergency University Hospital). Pre-operative biochemical evaluations and bacteriological examinations (urinary and internal stent sampling) were performed in D0, D2, D30 and D60 dynamics, on non-selective media (BCP = bromocresol purple lactose and CLED = cystine–lactose–electrolyte-deficient). RT-PCR tests were carried out for patients with suspected COVID-19 disease during hospitalization (14 patients were detected as being COVID-19-positive). A probabilistic antibiotic treatment was administered for 5 days (2 days Cefort and 3 days Levofloxacin) in the case of positive bacteriology. All collected data were statistically analyzed using IBM SPSS software.

### 2.2. Statistic Survey

#### 2.2.1. Survey Design and Research Sample

In order to fulfill the purpose of the research, a quantitative study was carried out using 108 urologists working in Romania. The research was carried out between January 2023 and February 2023. The sampling method that was used in this study was that of the snowball. The questionnaire was sent to the doctors that were working at this moment in some hospitals. After that, they were asked to forward the questionnaire to their colleagues (urological doctors) working in the same unit. The data collection was performed with the help of a questionnaire that was posted on an online platform and distributed to the respondents.

The first question was a filter and had the role of selecting only those who were part of the research community, namely the urologists in Romania. At the end of the questionnaire, there were several questions that had the role of building the profile of the respondents who participated in this study. In the questionnaire were also questions aimed at identifying the doctors who had installed internal stents so far, as well as the frequency with which they had installed such medical devices. In the questionnaire, there were also questions that had the role of presenting the experience of doctors with the lithiasis patients from a communication process perspective. Thus, through the questions from the questionnaire, we identified the communication difficulties encountered by doctors, the main problems caused by faulty communication and the opinion of the doctors regarding how new technologies can help them to improve the communication process with their patients.

The other questions had the role of fulfilling the purpose of the research. In order to measure the relationship between the variables, a 7-point Likert scale was used. The dependent variable at the level of the linear multiple regression model was the perception of physicians regarding the importance of using new technologies to improve the communication process with lithiasis patients treated via internal urinary drainage pre- and post-COVID-19 period. The independent variables analyzed at the level of the regression model were the advantages perceived by urologists regarding new technologies of online communication with lithiasis patients, the perceived disadvantages, the perceived risks, the current communication process with lithiasis patients, the experience of urologists in using new communication technologies, the trust of urologists in new online communication technologies, the empathy (openness of physicians) in using new online communication technologies, the security of new online communication technologies, the cost of new online communication technologies, the notoriety of new online communication technologies, the image of new technologies of online communication among urologists and patients’ access to new communication technologies, as well as the possibility of conducting training among patients regarding the use of these applications.

#### 2.2.2. The Hypotheses That Were the Basis of the Quantitative Study

**H1:**  *The advantages perceived by urologists regarding new online technologies have a direct and positive impact on physicians’ perception of the importance of using them to improve the communication process with lithiasis patients.*

**H2:**  *The disadvantages perceived by urologists regarding new online communication technologies have a direct and negative impact on physicians’ perception of the importance of using them to improve the communication process with lithiasis patients.*

**H3:**  *The risks perceived by urologists regarding new online communication technologies have a direct and negative impact on physicians’ perception of the importance of using them to improve the communication process with lithiasis patients.*

**H4:**  *The current communication process with lithiasis patients has a direct and negative impact on the perception of physicians regarding the importance of using new technologies to improve the communication process with lithiasis patients.*

**H5:**  *The experience of urologists in using new communication technologies has a direct and positive impact on physicians’ perception of the importance of using them to improve the communication process with lithiasis patients.*

**H6:**  *The confidence of urologists in new online communication technologies has a direct and positive impact on the perception of physicians regarding the importance of using them to improve the communication process with lithiasis patients.*

**H7:**  *The physicians’ empathy in using new online communication technologies has a direct and positive impact on the physicians’ perception of the importance of using them to improve the communication process with lithiasis patients.*

**H8:**  *The security of new online communication technologies has a direct and positive impact on physicians’ perception of the importance of using them to improve the communication process with lithiasis patients.*

**H9:**  *The cost of new online communication technologies has a direct and negative impact on physicians’ perception of the importance of using them to improve the communication process with lithiasis patients.*

**H10:**  *The notoriety of new online communication technologies has a direct and positive impact on physicians’ perception of the importance of using them to improve the communication process with lithiasis patients.*

**H11:**  *The image of new online communication technologies among urologists has a direct and positive impact on physicians’ perception of the importance of using them to improve the communication process with lithiasis patients.*

**H12:**  *Patients’ access to new communication technologies has a direct and positive impact on physicians’ perception of the importance of using them to improve the communication process with lithiasis patients.*

**H13:**  *The possibility of training in the use of new communication technologies has a direct and positive impact on the perception of physicians regarding the importance of using them to improve the communication process with lithiasis patients.*

## 3. Results

### 3.1. Clinical Study Results

The study group included 212 patients, and of which 127 were men (60%) and 85 were women (40%), with a sex ratio M/W of 1.49. The average age of the patients was 55 years old, with extremes of 19 and 80 years old. Fourteen patients were found to be COVID-19-positive during hospitalization, remaining asymptomatic. They presented hyperalgesic nephritic colic or calculi larger than 5 mm in diameter, which were non-pelvic/meatal-located, with little chance of spontaneous elimination. In the present study, the indication for the internal stent was complicated urolithiasis in 82% of the cases (174 patients) with uretero-hydronephrosis, bilateral in 39% of the cases (68 patients) and unilateral in 61% of the cases (106 patients). From the point of view of urinary stone localization, the distribution was as follows: 28% on the right (59 patients) and 72% on the left (153 patients); 52% pelvic ureteral stones (110 patients) and 48% pyelic lithiasis (102 patients).

A group of 98 patients (46%) presented acute kidney failure, with the consequence of ischemic parenchymal atrophy secondary to uretero-hydronephrosis, requiring the internal stent insertion in emergency mode. In the other 54% (114 patients), the act was performed via medical appointment. There was a spontaneous expulsion of the stone after the insertion of the internal stent in 95 patients with pelvic ureteral lithiasis (45% of the group), probably due to ureteral dilatation secondary to the presence of the endoprosthesis. Urine monitoring revealed positive bacteriology in 11% of the group (23 patients), pre-operatively on day 0 (D0) and post-operatively for 19.5% (41 patients) on the second day (D2), 28% (59 patients) on day 30 (D30) and 35% (74 patients) on day 60 (D60) (Table 1).

The study revealed an internal stent colonization rate of 39% (83 patients) versus a urinary colonization rate of 35% (74 patients). Eight COVID-19-positive patients presented urinary colonization, representing a rate of 58%.

Table 2 presents the different germs that were identified, with the predominance of *Escherichia coli*, *Klebsiella pneumoniae* and *Enterococcus faeccalis*, both at the urinary level and in the ureteral stents.

The monitoring of the bacteriological profile of the urine for the group of 129 patients with non-colonized internal stents showed that only 19 patients (15%) presented subsequent urinary colonization, and 85% (110 patients) remained urinary-non-colonized. In the group of patients with colonized internal stents (83 patients), only 15 (18%) remained bacteriologically negative throughout the study, and 68 patients (82%) had one or more episodes of urinary tract infection.

The colonization of ureteral internal stents plays an essential role in the pathogenesis of urinary infections, increasing their occurrence. In this study, 98 patients (46%) with urolithiasis had emergency internal stent insertion, and 62 (64%) of them had internal stents colonized at D60. The other 114 patients had the internal stent insertion carried out via appointment, and only 30 (27%) of them had colonized internal stents at D60.

### 3.2. Statistical Study Results

Analyzing from the perspective of the profile of the respondents, it should be stated that 4.6% of those who participated in the study were women, while 95.4% of the physicians were men. Regarding the distribution according to the age of the respondents, 49.1% of those who participated in the study were aged between 36 and 45, and 25.9% of them were aged between 46 and 55, while 25% of them were aged between 25 and 35. Regarding the institution where they worked, 79.6% of the urologists who participated in the study mainly worked in a state medical facility while 20.4% of them worked in the private system.

The results of the quantitative study illustrated the fact that all of the physicians who participated in the research had, up until now, mounted at least one internal stent. Regarding the frequency with which they resorted to this medical procedure, 10.2% of those who participated in the study stated that, daily, they were involved in an internal stent installation/extraction procedure, 24.1% of them stated that they used this medical technique 2–3 times a week, 32.4% of them specified the fact that they had patients who required such a procedure once a week and 20.4% of the respondents specified the fact that they used this medical technique 2–3 times a month. A smaller part of the respondents (13%) stated that they used this technique less often, more precisely once a month. Regarding the process of communication with lithiasis patients, 88% of those who participated in the study stated that they encountered difficulties because patients did not show up for appointments to replace or extract the mounted internal stent, which led to the appearance of certain complications. The main problems identified by urologists following poor communication with lithiasis patients were delays in changing internal stents (51.9%), delays in removing internal stents (22.2%) and balms regarding the prescribed treatments (12%), as well as a series of adverse effects that were not treated in time due to poor communication (13.9%) (Table 3).

All of these problems arising from poor communication with patients have led to the emergence of ailments among them. Thus, 50% of the urologists mentioned the fact that all of these caused damage to the kidney, 11.1% of them stated that some patients lost their kidneys because they did not come to a medical facility in time, 10.2% of the physicians specified the fact that all of the problems caused trouble in mounting a new internal stent and 1.9% of them specified the fact that some delays led to the impossibility of mounting a new internal stent. A total of 26.9% of those who participated in the study specified other medical problems that the patients faced because they did not come to a medical facility in time.

Regarding the extent to which new technologies could help urologists to improve their communication with patients and reduce the risks that may arise due to poor communication, 92.6% of those who participated in the study stated that new technologies have the ability to improve the communication process with lithiasis patients, while 7.4% of them stated that they were not so effective in solving this problem.

#### The Proposed Multiple Linear Regression Model

At the level of the quantitative research, a multiple linear regression model was created that aimed to determine how the independent variables at this level had the ability to influence the dependent variable. To analyze the multiple linear regression model, we used the analysis of variance method (ANOVA). This method helped us to identify the relationship between the dependent variables and the independent variables. The data were analyzed using the SPSS program.

The dependent variable analyzed at the level of the linear regression model was the physicians’ perception of the importance of using new online technologies to improve the communication process with lithiasis patients treated via internal urinary drainage pre- and post-COVID-19 period. The independent variables considered were as follows: the advantages perceived by urologists regarding new online communication technologies, the perceived disadvantages, the perceived risks, the current communication process with lithiasis patients, the experience of urologists in using new communication technologies, the confidence of urologists in new online communication technologies, the empathy (physicians’ openness) in using new online communication technologies, the security of new online communication technologies, the cost of new online communication technologies, the awareness of new online communication technologies, the image of new online communication technologies among urologists, patients’ access to new communication technologies and the possibility of training patients regarding the use of these technologies.

A multiple linear regression model was carried out, based on the following formula:

*Y* = *β*0 + *β*1 × *X*1 + *β*2 × *X*2 + *β*3 × *X*3 + *β*4 × *X*4 + … + *βn* × *Xn* + *Ɛ*

The components of this formula are as follows: *Y* (the dependent variable); *β*0 (the constant); *β*1 − *βn* (*β* coefficients for the independent variables); *X*1 − *Xn* (the model parameters estimation) and *Ɛ* (the standard error).

Starting from this formula, the multiple linear regression model was drawn up for the study:

*Physicians’ perception of the importance of using the new technologies to improve the communication process with lithiasis patients treated by internal urinary drainage before and post-COVID-19 period = β*0 + *β*1 × The advantages perceived by urologists regarding new online communication technologies with lithiasis patients + *β*2 × The perceived disadvantages + *β*3 × The perceived risks + *β*4 × The current communication process with lithiasis patients + *β*5 × The experience of urologists in the use of new communication technologies + *β*6 × The confidence of urologists in new online communication technologies + *β*7 × The empathy (physicians’ openness) in the use of new online communication technologies + *β*8 × The security of new online communication technologies + *β*9 × The cost of new online communication technologies + *β*10 × The notoriety of new online communication technologies + *β*11 × The image of new online communication technologies among urologists + *β*12 × Patients’ access to new communication technologies + *β*13 × The possibility of carrying out training among patients + *Ɛ*.

Regarding the reliability of the scale, it was calculated using Cronbach’s alpha coefficient. In this statistical study, the indicator was 0.746 > 0.7, highlighting in this way the viability of the variables that was taken into consideration in the linear model regression (Table 4).

Regarding the results obtained at the level of the linear multiple regression model, it should be stated that the value of R is 0.775 while that of R square is 0.600 (Table 5). This aspect illustrates the fact that 60% of the variation in physicians’ perception regarding the importance of using new technologies to improve the communication process with lithiasis patients treated via internal urinary drainage pre- and post-COVID-19 period is explained by the independent variables that were taken into account at the level of the model.

When analyzing from the perspective of the validity of the model, it can be observed that the value of Sig. is 0.000. Since this value is less than 0.05, the model is considered to be a valid one. From the table above (Table 6), it can be seen that the standard error has a value of 1.056, while the value of F is 10.862. At the analysis level, there are 13 degrees of freedom (df) and the mean square value is 12.104 (Table 6).

In the table of coefficients (Table 7), it can be seen that not all of the independent variables can be taken into account at the level of the multiple linear models, because the value of Sig. for these is greater than 0.05. The variables that were not taken into account were the security of new online communication technologies, the cost of new online communication technologies, the notoriety of new online communication technologies, the image of new communication technologies among urologists, the access of patients to new communication technologies and the possibility of carrying out training among patients regarding the use of these new technologies.

Based on the results obtained previously, the formula of the linear multiple regression model is as follows:

*Physicians’ perception of the importance of using the new technologies to improve the communication process with lithiasis patients treated by internal urinary drainage pre- and post-COVID-19 period* = 7.182 + 0.785 × The advantages perceived by the urologists regarding new communication technologies with lithiasis patients was − 0.666 × The perceived disadvantages were − 0.265 × The perceived risks were − 0.206 × The current communication process with lithiasis patients was + 0.341 × The experience of urologists in the use of new communication technologies was + 0.250 × The confidence of urologists in new online communication technologies was + 0.586 × The empathy (physicians’ openness) in the use of new online communication technologies was + 1.056.

## 4. Discussion

The COVID-19 pandemic has caused a series of problems for both physicians and patients. The restrictions imposed at the national level as well as the fear of infection with the new virus led patients to be more fearful and to cancel the appointments that they had, even though they suffered from certain conditions that required urgent interventions. For lithiasis patients, this was a real problem, as delays in changing or extracting the internal stents led to complications, which caused physicians to face some difficulty in solving these problems. Considering these things, it must be stated that new technologies have a very important role in terms of improving the communication process with lithiasis patients. Thus, e-health [30], mobile health [31] and telemedicine [32,33] are just some of the technologies that can be used to improve the healthcare industry [34]. Healthcare providers around the world are constantly adopting various technologies to meet increasing regulatory requirements for patient care and safety. In addition, they address two important perspectives: the growing need to reduce healthcare costs as well as the need to continuously improve the quality-of-care services, while maintaining the operational efficiency of healthcare organizations [35].

Communication systems can be used to fulfill several objectives related to aspects of diagnosis, management, counseling, education and support. They can be applied to facilitate communication between healthcare professionals or between professionals and patients. There is a wide range of communication systems, from e-mail and mobile phones to telemedicine and telehealth systems. Decision support systems are automated systems accessible from various devices such as computers, mobile phones or personal digital assistants (PDAs) [36]. They support decision-making for healthcare professionals and help them to practice various activities in accordance with clinical guidelines and care plans. Information systems, such as web-based resources and e-health portals, refer to the use of Internet technology to access medical information sources [37].

The term eHealth [38,39] can describe “a technical development, a state of mind, a way of thinking, an attitude to improve the health sector at the local, regional and global level through the use of information and communication technology” [40,41]. The current trend in eHealth systems is to focus on the patient. Thus, the patient actively takes part in the management of his/her health, being supported by a dynamic environment of innovative services aimed at improving access to care. eHealth [42] focuses on prevention and empowering consumers to proactively manage their own health. From the healthcare provider’s perspective, eHealth increases efficiency by decreasing hospitalizations and the length of stay.

In order for a digitalization approach to be successful, existing healthcare practices need to be streamlined, simplified and redesigned, as they need to be thought about differently from the traditional paper-based system [43]. Telemedicine encompasses “examinations, monitoring and treatment, as well as educational sessions, processed using ICT-based systems that allow direct access to experts and patient information, regardless of location” [44]. Telemedicine [45] can be applied using any media and connection technology, such as video communications, e-mail, electronic monitoring equipment and web portals [46]. The definition provided by the American Telemedicine Association is “the use of medical information exchanged from one site to another through electronic communications in order to improve the clinical health status of the patient, including applications and services that use two-way video, e-mail, smartphones, wireless tools and other forms of telecommunication technology” [47].

At this moment, consumers’ preferences regarding the use of digital technologies in the medical field are not fully known. Patients suffering from more serious conditions such as cancer consider new medical technologies as a means of high-level information but also as a form of support in decision-making [48]. More work is needed to identify barriers to access, especially for patients with severe conditions, who also have other financial or educational problems [49]. If digital technologies in the medical field are not implemented based on careful strategic planning, which takes into account existing barriers, supporting factors, needs and opportunities for patient involvement, they can cause negative effects regarding their implementation [50,51].

The COVID-19 pandemic has had important medical, social and economic repercussions and has required an unprecedented relocation of human, hospital, financial and research means worldwide [52]. Urologists were forced to undertake joint urological practices with health imperatives related to COVID-19 (patients held in isolation, modification of the operating schedule, prioritization of some procedures, rescheduling consultations). The COVID-19 pandemic had a very strong impact on the communication process between patients and doctors. The restrictions imposed at the national level as well as the fear of infection with the new virus have led patients to be more reserved in terms of making appointments in hospitals. The development of the Internet and information and communication technologies [53] in the recent period of time has had a very important role, with studies illustrating the fact that they contribute, to an important extent, both to increasing the productivity of physicians [54] and to the degree of satisfaction felt by patients.

The clinical study illustrates that the emergency insertion of ureteral prostheses showed a 64% prevalence of internal stent colonization in D60, which is significantly higher than scheduled insertion (27%). The prevalence of urinary colonization increases with the indwelling time of ureteral prostheses from 11% to 35% at D60. Due to the risk of post-COVID-19 complications, the monitoring of the kidney function for the 14 patients diagnosed as being COVID-19-positive will be needed.

The quantitative study that was carried out among urologists in Romania illustrated the fact that they frequently carry out installation activities of internal stents for patients with kidney diseases, with a large part of them carrying out such interventions weekly (32.4%). The results illustrated the fact that 88% of the respondents encountered difficulties in the communication process with stone patients during the COVID-19 pandemic, but also after its end. A total of 51.9% of urologists stated that poor communication between them and patients led to delays in changing internal stents, while 22.2% of them stated that poor communication led to a delay in the extraction of internal stents. All of these aspects automatically caused the appearance of medical problems among patients, with 50% of the urologists mentioning the fact that these delays caused damage to the patients’ kidneys to a certain extent, and 10.2% of them stating that they encountered subsequent difficulties in fitting a new internal stent. A total of 11% of the respondents specified the fact that there were also situations where, due to poor communication, patients did not show up in time to change or extract their internal stent, a fact that even led to the loss of the kidney. A large part of those who participated in the study (92.6%) considered that new online technologies could bring added value to the communication process with lithiasis patients. After analyzing the linear regression model, it was noticed that the initially proposed model is a valid one, with the recorded value of Sig. being 0.000 < 0.05. In addition, it was noticed that the variation in the dependent variable at the level of the multiple linear regression model was explained in the proportion of 60% by the independent variables that were taken into account.

Regarding the strengths of the clinical study, it has offered a lot of information regarding the impact that the SARS-CoV-2 virus had on lithiasis patients, it has provided valuable information regarding the risk of urinary colonization for these patients who have internal stents and the study has also provided information regarding the post-COVID-19 complications that can occur for these patients. The strengths of the statistical study are as follows: the study provides an overall picture of the perception of urologists regarding the importance of using new technologies to improve communication with lithiasis patients and it illustrates the main problems that can be solved through the use of these technologies, as well as the factors that have the ability to influence the perception of physicians regarding the use of these technologies.

Regarding the weaknesses of the clinical study that was carried out, it must be stated that it was conducted on a limited number of respondents (212). In order to have a broader picture of the studied problem, larger studies should be conducted to certify the findings identified on a larger scale. Regarding the weaknesses of the quantitative research, it must be stated that a first aspect refers to the limited number of respondents who participated in the study. In addition, at this level, only a few of the variables that have the ability to influence the perception of physicians regarding the importance of using new technologies to facilitate the communication process with lithiasis patients were taken into account.

In the future, in order to better understand the studied topic, in-depth interviews should be conducted with urologists in Romania to better understand how online communication technologies can be used in this field, in order to facilitate communication with lithiasis patients. In addition, certain focus groups should also be conducted with patients, to observe the extent to which they would agree to use certain applications or technologies if it meant communicating with urologists more easily, and to find out, in real time, the treatments that they need, and the period in which they should see a physician to change or remove the internal stents that they have. In addition, future studies based on this topic should be conducted in both private and public health facilities to observe whether there are differences in the communication process at the levels of the two types of institutions. All of these aspects can create a broader picture of the presented topic, highlighting the most appropriate way in which online communication technologies can be used to facilitate communication with lithiasis patients.

## 5. Conclusions

Disruptive technologies can be considered to be an optimal solution for solving the communication problem. Better communication between stone patients and urologists would ensure strict adherence to appointments and allow specialists to learn the subsequent reactions that patients have, in real time. The clinical study illustrates that for patients with urolithiasis indwelling internal stents, 39% was the prevalence of colonized internal stents and 35% was the prevalence of urinary colonization (58% in the case of those who were COVID-19-positive). In patients with non-colonized internal stents, the incidence of urinary colonization was 15%, while 82% of the patients with colonized internal stents were also urinary-colonized, presenting one or more episodes of urinary infection. The risk of urinary colonization is significantly higher in patients with already-colonized ureteral internal stents. In the statistical study, we noticed that the perception of physicians regarding the importance of using new technologies to improve the communication process with lithiasis patients treated via internal urinary drainage pre- and post-COVID-19 period is directly and positively influenced by the following: the advantages perceived by urologists with regard to new technologies of online communication with lithiasis patients, the experience of urologists in the use of new communication technologies, the trust of urologists in new technologies of online communication and the openness of physicians in the use of new technologies at the level of the communication process, and that it is influenced in a negative way by the following: the disadvantages perceived by urologists regarding new technologies of online communication with lithiasis patients, the perceived risks and the current communication process with lithiasis patients. The results obtained in this paper present increased importance for doctors, patients and hospital managers. The hospital managers can identify the perception of the doctors regarding how new communication technologies can be used in hospitals to improve the communication process with patients. In addition, they have valuable information regarding the importance given by doctors to certain factors when they want to improve the communication process with lithiasis patients. Starting from these results, a series of measures can be taken both within state hospitals and within private clinics to implement new methods of communication with these patients, taking into account the influencing factors mentioned in this study.

## Figures and Tables

**Table 1:** Positive urinary bacteriology.

Number of Days of Double Internal Stent Insertion	Positive Urinary Bacteriology	
	Number of Patients	%
D_0_	23	11
D_2_	41	19.5
D_30_	59	28
D_60_	74	35

Source: clinical study conducted by the authors.

**Table 2:** The bacterial germs identified at the urinary level and in the ureteral stents.

Bacterial Germ	Number of Patients	
	Urinary-Colonized	Internal-Stent-Colonized
*Escherichia coli*	23	31
*Klebsiella pneumoniae*	17	13
*Enterococcus faecalis*	14	16
*Enterobacter cloaecae*	2	4
*Serratia marcescens*	2	0
*Staphylococcus hominis*	5	0
*Staphylococcus aureus*	2	10
Others	9	9

Source: clinical study conducted by the authors.

**Table 3:** Problems arising from poor communication with lithiasis patients.

Outcomes of Poor Communication with Lithiasis Patients	Frequency	Percentage (%)
Delays in changing internal stents	56	51.9
Delays in the extraction of the internal stents	24	22.2
Problems regarding prescribed treatments	13	12.0
The occurrence of adverse effects following the installation of the internal stents	15	13.9

Source: statistical study conducted by the authors.

**Table 4:** Reliability statistics.

Cronbach’s Alpha	Cronbach’s Alpha Based on Standardized Items	N of Items
0.746	0.759	13

Source: statistical study conducted by the authors.

**Table 5:** Indicators of the multiple linear regression model.

Indicators	Validation Criteria
R	0.775
R Square	0.600
Adjusted R Square	0.545
Std. Error of the Estimate	1.056
R Square Change	0.600
F Change	10.862
df1	13
df2	94
Sig. F Change	0.000

Source: statistical study conducted by the authors.

**Table 6:** ANOVA table.

Model	Sum of Squares	df	Mean Square	F	Sig.
Regression	157.353	13	12.104	10.862	0.000
Residual	104.749	94	1.114		
Total	262.102	107			

Source: statistical study conducted by the authors.

**Table 7:** Table of coefficients.

Model	Unstandardized Coefficients		Standardized Coefficients	Sig.
	B	Std. Error	Beta	
(Constant)	7.182	1.029		0.000
The security of new online communication technologies	−0.030	0.109	−0.029	0.786
The current communication process with lithiasis patients	−0.224	0.110	−0.206	0.045
The cost of new online communication technologies	0.013	0.111	0.012	0.909
The notoriety of new online communication technologies	−0.090	0.070	−0.106	0.204
The risks perceived by urologists	−0.305	0.131	−0.265	0.022
The advantages perceived by urologists	0.867	0.194	0.785	0.000
The image of new communication technologies among urologists	−0.175	0.183	−0.150	0.343
The experience of urologists in the use of new communication technologies	0.342	0.147	0.341	0.022
The disadvantages perceived by urologists	−0.742	0.201	−0.666	0.000
Patients’ access to new online communication technologies	−0.278	0.185	−0.176	0.136
The confidence of urologists in new online communication technologies	0.335	0.157	0.250	0.035
The possibility of carrying out training for lithiasis patients	−0.242	0.134	−0.195	0.075
Physicians’ empathy toward new online communication technologies	0.577	0.123	0.586	0.000

Source: statistical study conducted by the authors.

## Data Availability

Not applicable.

## References

[1] World Health Organization Tuberculosis (TB): Frequently Asked Questions on Global Task Force on Digital Health for TB and Its Work. Geneva, Switzerland. https://www.who.int/tb/areas-of-work/digital-health/faq/en/.

[2] Huckvale C., Car J., Akiyama M., Jaafar S., Khoja T., Khalid A.B., Sheikh A., Majeed A. (2010). Information technology for patient safety. Qual. Saf. Health Care.

[3] Lera M., Taxtsoglou K., Iliadis C., Frantzana A., Kourkouta L. (2020). The Use of New Information and Communication Technologies in Nursing Practice. EAS J. Nurs. Midwifery.

[4] Mair F., May C., Murray E., Finch T., Anderson G., O’Donnell C., Wallace P. (2009). Understanding the Implementation and Integration of e-Health Services-Research Report Produced for the NIHR SDO Program. http://www.netscc.ac.uk/hsdr/files/project/SDO_FR_08-1602-135_V01.pdf.

[5] Catan G., Espanha R., Mendes R., Toren O., Chinitz D. (2015). The Impact of eHealth and mHealth on doctor behavior and patient involvement: An Israeli and Portuguese comparative approach. Stud. Health Technol. Inform..

[6] Kichloo A., Albosta M., Dettloff K., Wani F., El-Amir Z., Singh J., Aljadah M., Chakinala R.C., Kanugula A.K., Solanki S. (2020). Telemedicine, the current COVID-19 pandemic and the future: A narrative review and perspectives moving forward in the USA. Fam. Med. Community Health.

[7] Scheffer M., Cassenote A., de Britto e Alves M.T.S.S., Russo G. (2022). The multiple uses of telemedicine during the pandemic: The evidence from a cross-sectional survey of medical doctors in Brazil. Glob. Health.

[8] Istepanian R.S.H. (2022). Mobile Health (m-Health) in Retrospect: The Known Unknowns. Int. J. Environ. Res. Public Health.

[9] Ku J.P., Sim I. (2021). Mobile Health: Making the leap to research and clinics. npj Digit. Med..

[10] Agarwal S., Perry H.B., Long L.-A., Labrique A.B. (2015). Evidence on feasibility and effective use of mHealth strategies by frontline health workers in developing countries: Systematic review. Trop. Med. Int. Health.

[11] Wyatt J.C., Sullivan F. (2005). eHealth and the future: Promise or peril?. BMJ.

[12] Abdulrahman A.A., Iason K., Panagiotis K. (2010). Ureteral stents: New ideas, new designs. Adv. Urol..

[13] Bouzidi H., Traxer O., Doréc B. (2008). Caractéristiques des incrustations des endoprothèses urétérales chez les patients lithiasiques. Prog. Urol..

[14] Hubner W.A., Plas E.G., Stoller M.L. (1992). The double-J ureteral stent: In vivo and in vitro flow studies. J. Urol..

[15] Finney R. (1978). Experience with next double J ureteral catheter stent. J. Urol..

[16] Goldfischer E.R., Gerber G.S. (1997). Endoscopic management of ureteral strictures. J. Urol..

[17] Akay A.F., Aflay U., Gedik A., Sahin H., Bircan M.K. (2007). Risk factors for lower urinary tract infection and bacterial stent colonisation in patients with a double J ureteral stent. Int. Urol. Nephrol..

[18] Paick S.H., Park H.K., Oh S.J., Kim H.H. (2003). Characteristics of bacterial colonization and urinary tract infection after indwelling of double-J stent. Urology.

[19] Riedl C.R., Plas E., Hubner W.A. (1999). Bacterial colonization of ureteral stents. Eur. Urol..

[20] Cohen S.N., Kass E.H. (1967). A simple method for quantitative urine culture. N. Engl. J. Med..

[21] Yeniyol C.O., Tuna A., Yener H., Zeyrek N., Tilki A., Coskuner A. (2002). Bacterial colonization of double J stents and bacteriuria frequency. Int. Urol. Nephrol..

[22] Petriconi R., Zores T. (2014). Dérivation du haut appareil urinaire par sonde urétérale, double J, néphrostomie ou pontage interne: Principes, techniques et complications. EMC Tech. Chir. Urol..

[23] Türk C., Petřík A., Sarica K., Seitz C., Skolarikos A., Straub M., Knoll T. (2016). EAU Guidelines on Interventional Treatment for Urolithiasis. Eur. Urol..

[24] Daifuku R., Stamm W.E. (1986). Bacterial adherence to bladder uroepithelial cells in catheter-associated urinary tract infection. N. Engl. J. Med..

[25] Jones R., Young P., Marasseky J. (1982). Treatment of infection in the presence of an indwelling urethral catheter. Br. J. Urol..

[26] Comité Technique National des Infections Nosocomiales 100 Recommandations Pour la Surveillance et la Prévention des Infections Nosocomiales. Ministère de l’Emploi et de la Solidarité- Secrétariat d’Etat à la Santé et à l’Action Sociale. Deuxième Édition 1999. https://medias.vie-publique.fr/data_storage_s3/rapport/pdf/014000029.pdf.

[27] Garcia-Martin M., Lardelli-Claret P., Jimenez-Moleon J.J., Bueno-Cavanillas A., Luna-del-Castillo J.D., Galvez-Vargas R. (2001). Proportion of hospital deaths potentially attributable to nosocomial infection. Infect. Control Hosp. Epidemiol..

[28] Marty L., Jarlier V. (1999). Surveillance des bactéries multirésistantes. Prog. Urol..

[29] Nickel J.C., Costerton J.W., McLean R.J., Olson M. (1994). Bacterial biofilms: Influence on the pathogenesis, diagnosis and treatment of urinary tract infections. J. Antimicrob. Chemoth..

[30] Wang W., Sun L., Liu T., Lai T. (2022). The use of E-health during the COVID-19 pandemic: A case study in China’s Hubei province. Health Sociol Rev..

[31] Degavre F., Kieffer S., Bol D., Dekimpe R., Desterbecq C., Pirson T., Sandu G., Tubeuf S. (2022). Searching for Sustainability in Health Systems: Toward a Multidisciplinary Evaluation of Mobile Health Innovations. Sustainability.

[32] Ravindrane R., Patel J. (2022). The environmental impacts of telemedicine in place of face-to-face patient care: A systematic review. Future Healthc. J..

[33] Amjad A., Kordel P., Fernandes G. (2023). A Review on Innovation in Healthcare Sector (Telehealth) through Artificial Intelligence. Sustainability.

[34] Escobar O., Leone D., Malafronte P., Mele S. (2021). The Effect of Telemedicine on Patients’ Wellbeing: A Systematic Review. Int. J. Econ. Manag..

[35] Hammer R. 15 Amazing Healthcare Technology Innovations. https://getreferralmd.com/2016/01/healthcaretechnology-2016/.

[36] Balas E.A., Boren S.A., Brown G.D. (2000). Studies in Health Technology and Informatics.

[37] Rouleau G., Gagnon M.P., Côté J., PayneGagnon J., Hudson E., Dubois C.A. (2017). Impact of Information and Communication Technologies on Nursing Care: Results of an Overview of Systematic Reviews. J. Med. Internet Res..

[38] da Fonseca M.H., Kovaleski F., Picinin C.T., Pedroso B., Rubbo P. (2021). E-Health Practices and Technologies: A Systematic Review from 2014 to 2019. Healthcare.

[39] Wang H., Yu X. (2022). A Review of e-Health Research in Information System. J. Biomed. Sci. Eng..

[40] Eysenbach G., Diepgen T. (2001). The role of e-health and consumer health informatics for evidence-based patient choice in the 21st century. Clin. Dermatol..

[41] Boogerd E.A., Arts T., Engelen L.J., van de Belt T.H. (2015). “What Is eHealth”: Time for An Update?. JMIR Res. Protoc..

[42] Mauco K.L., Scott R.E., Mars M. (2020). Validation of an e-health readiness assessment framework for developing countries. BMC Health Serv. Res..

[43] Pohlmann S., Kunz A., Ose D., Winkler E.C., Brandner A., Poss-Doering R., Szecsenyi J., Wensing M. (2020). Digitalizing Health Services by Implementing a Personal Electronic Health Record in Germany: Qualitative Analysis of Fundamental Prerequisites From the Perspective of Selected Experts. J. Med. Internet Res..

[44] Tsioumanis V., Mangita A., Diomidous A. (2016). Applications and developments of telemedicine in Greece. Unifying the Applications and Foundation of Biomedical and Health Informatics. Stud. Health Technol. Inform..

[45] Payan D.D., Frehn J.L., Garcia L., Tierney A.A., Rodriguez H.P. (2022). Telemedicine implementation and use in community health centers during COVID-19: Clinic personnel and patient perspectives. SSM Qual. Res. Health.

[46] Su Z., Li C., Fu H., Wang L., Wu M., Feng X. (2022). Review of the development and prospect of telemedicine. Intell. Med..

[47] Glinkowski W.M., Karlińska M., Karliński M., Krupiński E.A. (2018). Telemedicine and eHealth in Poland from 1995 to 2015. Adv. Clin. Exp. Med..

[48] Cooley M.E., Nayak M.M., Abrahm J.L., Braun I.M., Rabin M.S., Brzozowski J., Lathan C., Berry D.L. (2017). Patient and caregiver perspectives on decision support for symptom and quality of life management during cancer treatment: Implications for eHealth. Psychooncology.

[49] Gonzalez B.D. (2018). Promise of mobile health technology to reduce disparities in patients with cancer and survivors. JCO Clin. Can. Inform..

[50] Bradford N., Caffery L., Smith A. (2016). Telehealth services in rural and remote Australia: A systematic review of models of care and factors influencing success and sustainability. Rural Remote Health.

[51] Rossen S., Kayser L., Vibe-Petersenm J., Ried-Larsen M., Christensen J.F. (2019). Technology in exercise-based cancer rehabilitation: A cross-sectional study of receptiveness and readiness for e-Health utilization in Danish cancer rehabilitation. Acta Oncol..

[52] Gheorghiță V., Guță-Alexandru L. (2022). Pandemia de COVID-19 în Romania Modalitățile de Răspuns și Rolul Rezilienței Psihologice în Situații de Criză–Studiu de Caz.

[53] Radu A.V., Tascu A.V., Stoica I., Radu A.C., Purcarea V.L. (2017). Online instruments used in pharmaceutical marketing. Farmacia.

[54] Stoica (Răpan) I., Zaman G., Suciu M.-C., Purcărea V.-L., Jude C.-R., Radu A.-V., Catană A., Radu A.-C. (2022). A Better Integration of Industrial Robots in Romanian Enterprises and the Labour Market. Appl. Sci..

